# Characteristics of Resting State EEG Power in 80+-Year-Olds of Different Cognitive Status

**DOI:** 10.3389/fnagi.2021.675689

**Published:** 2021-08-11

**Authors:** Stephanie Fröhlich, Dieter F. Kutz, Katrin Müller, Claudia Voelcker-Rehage

**Affiliations:** ^1^Department of Neuromotor Behavior and Exercise, Institute of Sport and Exercise Sciences, Faculty of Psychology and Sport Sciences, University of Münster, Münster, Germany; ^2^Department of Sports Psychology (With Focus on Prevention and Rehabilitation), Institute of Human Movement Science and Health, Faculty of Behavioural and Social Sciences, Chemnitz University of Technology, Chemnitz, Germany; ^3^Institute of Human Movement Science and Health, Faculty of Behavioural and Social Sciences, Chemnitz University of Technology, Chemnitz, Germany; ^4^Department of Social Science of Physical Activity and Health, Institute of Human Movement Science and Health, Faculty of Behavioural and Social Sciences, Chemnitz University of Technology, Chemnitz, Germany

**Keywords:** aged 80 and over, EEG reactivity, resting state EEG, eyes open, eyes closed, mild cognitive impairment

## Abstract

Compared with healthy older adults, patients with Alzheimer's disease show decreased alpha and beta power as well as increased delta and theta power during resting state electroencephalography (rsEEG). Findings for mild cognitive impairment (MCI), a stage of increased risk of conversion to dementia, are less conclusive. Cognitive status of 213 non-demented high-agers (mean age, 82.5 years) was classified according to a neuropsychological screening and a cognitive test battery. RsEEG was measured with eyes closed and open, and absolute power in delta, theta, alpha, and beta bands were calculated for nine regions. Results indicate no rsEEG power differences between healthy individuals and those with MCI. There were also no differences present between groups in EEG reactivity, the change in power from eyes closed to eyes open, or the topographical pattern of each frequency band. Overall, EEG reactivity was preserved in 80+-year-olds without dementia, and topographical patterns were described for each frequency band. The application of rsEEG power as a marker for the early detection of dementia might be less conclusive for high-agers.

## Introduction

Dementia is diagnosed due to pronounced cognitive impairments and deterioration in daily living, but pathophysiological changes in the brain usually occur before this critical stage is reached (Sperling et al., 2011). Mild cognitive impairment (MCI), which is characterized as objective cognitive deficits that are more severe than normal aging would suggest, but mild enough to not interfere with daily independence, is thought to be a precursor to dementia (Winblad et al., 2004). Older adults (OA) with MCI have a higher risk of developing dementia, particularly Alzheimer's disease (AD), compared to healthy OA (Mitchell and Shiri-Feshki, 2009) and show more brain neuropathology linked to dementia in postmortem studies (Petersen et al., 2006) and in studies with cerebrospinal fluid analysis (Visser et al., 2009). In longitudinal examinations, the development of patients with MCI is heterogeneous. For example, it was reported that 14% of MCI cases reverted back to normal cognition, 35% progressed to dementia, and 51% stayed stable at the 2-year follow-up (Pandya et al., 2017).

To further understand MCI and its progression to dementia, it is, important to study brain changes in MCI directly and to find biomarkers that better predict progression to dementia. Resting state electroencephalogram (rsEEG) measures seem to be especially suitable because they are easily obtained (non-invasive, no special stimuli necessary, short recording time) and can help to understand the connectivity of brain networks (Babiloni et al., 2019). Differences in rsEEG activity in eyes closed (EC) conditions between healthy OA and patients with AD have been shown consistently (in cross-sectional and longitudinal studies) and include decreased alpha and beta power, increased delta and theta power, and changes in coherence and other functional connectivity measures [for reviews, see Jeong (2004) and Babiloni et al. (2016)]. Similar results were reported for vascular dementia (van Straaten et al., 2012) while frontotemporal dementia does not show consistent differences in rsEEG compared with healthy OA (Nardone et al., 2018).

In contrast, only a few studies compared the rsEEG of healthy OA and OA with MCI during EC. The following studies all included the frequency bands delta, theta, alpha, and beta and reported inconsistent results. For example, in two cross-sectional studies from the same research group, MCI patients (age ~72 years) had less alpha 1 (8–10.5 Hz) power and stronger delta power, while no changes were present in the theta and beta bands (Babiloni et al., 2006b, 2010). Others also reported higher delta power in MCI (age, 71.9 ± 7.9 years) compared to healthy individuals of the same age and no significant differences in the other frequency bands (Ya et al., 2015). Alternatively, it was reported that theta power was decreased in OA with mild cognitive deficits (age, 70.7 ± 8.8 years) and that changes in other bands were present only in further cognitively declined groups (Prichep et al., 1994). Another study with participants of similar age in the MCI group (72.5 ± 6.0 years) reported lower delta and theta band power, but no change in the faster frequency bands (Kwak, 2006). This study included comparable fewer cases of MCI (*n* = 16) than all other studies mentioned, where the sample size for MCI ranged from 40 to 155 cases. In a different sample with a similar small MCI case amount (*n* = 20, age 74 ± 5 years), no significant differences between patients with MCI and healthy OA in the theta band were detectable, although theta power of the MCI group fell in-between healthy and OA with dementia (van der Hiele et al., 2007a). In addition, it was shown that patients with MCI (mean age, 70.7 years) revealed less alpha and less beta phase-locked synchronization (measured with global field synchronization instead of power), but no changes in the slower frequency bands (Koenig et al., 2005).

Taken together, no conclusive picture for the typical delta, theta, alpha, and beta power values during rsEEG in EC condition in the presence of MCI can be obtained from these studies. It seems that the direction of changes is comparable to findings in dementia. However, which of these changes are earliest in the transition toward dementia and, therefore, most common in MCI is unclear. This might be due to the limited number of studies, including preclinical stages of dementia, small sample sizes, heterogeneity in MCI classification, and heterogeneity in the underlying cause of MCI (Yang et al., 2019).

Heterogeneity of underlying causes for MCI also means that only a certain proportion of MCI cases will progress toward dementia and, therefore, might be the only ones displaying rsEEG patterns similar to those known in dementia. Moreover, many types of dementia exist with AD being the most common cause. Longitudinal studies can take this into account and examine which EEG power parameters at the baseline best predict further cognitive decline or even progression to AD or other types of dementia in OA with MCI. For example, posterior alpha power was reported to be smaller in progressing MCI compared with stable MCI cases (age of all MCI cases at the baseline, 65.9 ± 9.6 years) and predicted worsening of cognitive function in a 1-year period with 75% positive predictive power (Luckhaus et al., 2008). For a longer follow-up period of 21 months, one study has shown that relative alpha power, relative theta power, and mean frequency at the temporo-occipital region in EC conditions at the baseline (age at baseline, 58.2 ± 5.9 years) were the best EEG predictors for conversion to AD (Jelic et al., 2000). Accuracy of prediction was raised from only 70%, which was obtained with MMSE as the only predictor, to 85% by adding EEG parameters (Jelic et al., 2000). The best choice of parameters to predict conversion from MCI (age at the baseline, 68.7 years) to AD over a 2-year follow-up period obtained by data mining from 177 EEG parameters included predominantly beta frequency parameters and reached 88% sensitivity, 82% specificity, and 64% positive predictive value (Poil et al., 2013). The classification rates in all studies so far were not sufficient enough for diagnostic application (Jelic and Kowalski, 2009; Rossini et al., 2020).

Different causes for MCI also mean that subtypes of MCI should be differentiated. Most commonly, this is done by distinguishing between amnestic (aMCI) and non-amnestic (naMCI) cognitive deficits (Petersen, 2004). The aMCI is thought to be primarily related to AD because the relative incidence of AD is significantly higher in aMCI compared with naMCI, although other outcomes, such as vascular dementia or mixed forms, are also possible (Jungwirth et al., 2012). In addition, it has been shown that the amnestic subtype of MCI differs from the non-amnestic type and shows lower central alpha and greater occipital theta power at rest compared with naMCI (Babiloni et al., 2010). Magnetic resonance imaging (MRI) results also support the notion that neuropathological changes are different in both types (Guan et al., 2017).

In addition to disease-related changes, EEG oscillations at rest are also subject to changes during healthy aging. Research on rsEEG (mostly during EC) in healthy OA consistently reveals changes in the alpha band, which are similar to changes found in AD, such as reduced power and reduced peak frequency with increasing age (Rossini et al., 2007). For delta and theta bands, decreases were mostly reported (Babiloni et al., 2006a; Gaál et al., 2010), while activity in the beta band seems to be more pronounced in OA compared with young adults (Koyama et al., 1997; Rossiter et al., 2014). Those changes in delta, theta, and beta bands are in the opposite direction of those reported due to AD. Research on healthy OA as well as MCI, however, has mainly been conducted within the age range of 60–80 years. Thus, there seem to be no detailed reports about topographical or frequency specific EEG power characteristics in high-agers (>80 years) during rest or in comparison with younger OA.

Most studies so far only analyzed rsEEG data obtained while eyes were closed. Studying eyes open (EO) conditions seems appropriate, considering that task-related brain activity is dependent on the prior background activity (Başar and Güntekin, 2012), and cognitive tasks in everyday life are usually not solved in EC conditions. It has been shown that the classifications between healthy OA and MCI work better with data from EO than EC conditions (McBride et al., 2014). For example, alpha activity during EO was reduced in MCI compared with healthy OA, but alpha activity in EC was not able to discriminate between both groups (McBride et al., 2014). Including both conditions makes it possible to study states of low and moderate vigilance (Babiloni et al., 2019) and to differentiate between global arousal and focal activations (Barry et al., 2007). Investigating the changes from EC to EO conditions, termed EEG reactivity, might be promising as well. EEG reactivity describes the power difference in a frequency band between two distinct conditions (Klimesch, 1999). In the following, reactivity will be defined as the difference in power between EO rest and EC rest (EO-EC). Findings for reactivity are often limited to the alpha band. Synchronous alpha activity observed during EC is blocked when eyes are opened, which can be easily detected in the raw data (Berger, 1929). Healthy OA showed decreased alpha reactivity compared with young adults (Duffy et al., 1984) or a lack of reactivity at all (Gaál et al., 2010). Alpha reactivity was found to be even more decreased in patients with AD compared with healthy OA (van der Hiele et al., 2007b; Schumacher et al., 2020). In a study with small samples sizes, values of the MCI group (*n* = 11) were between the healthy (*n* = 12) and demented group (*n* = 10), but did not differ significantly from the healthy control group (van der Hiele et al., 2007b). Alpha reactivity was also found to be the best predictor of global cognitive performance, memory and language skills across all groups (van der Hiele et al., 2007b).

Recently, Barry and De Blasio (2017) have published rsEEG data for young adults (age, 20.4; range, 18.8–25.6 years) and OA (age, 68.2; range, 59.8–74.8 years), which looked in detail at the topographical characteristics of each frequency band and the changes from EC to EO conditions (reactivity) not only in the alpha frequency but also in the delta, theta, and beta bands. Across both groups, delta and theta power in EO and EC were midline dominant with a maximum at the vertex and a bias toward the right hemisphere (Barry and De Blasio, 2017). For the alpha band, the well-known posterior dominance was reported, and power in the right hemisphere was stronger compared with the left. Activation in the beta band showed centroparietal dominance. For young adults, changes from EO to EC included the overall reduction in power for delta, theta, alpha, and beta bands and a focal frontal increase in the beta frequency (Barry et al., 2007). A similar pattern was found in healthy OA, indicating that the EEG reactivity for delta, theta, alpha, and beta is maintained in healthy aging (Barry and De Blasio, 2017). No further studies exist that investigated EEG reactivity in other frequency bands than alpha in MCI or dementia.

From the current state of research, it can be concluded that further studies with adequate sample sizes are needed to better consider healthy aging as a reference point and the transition to cognitive decline (Yang et al., 2019), especially data for the oldest (>80 years) are lacking for neuropsychological as well as neurophysiological parameters (Slavin et al., 2013). Similarly, dementia research should include more of the oldest participants as they also make up the majority of the affected patients (Brayne and Davis, 2012; Richard et al., 2012).

The aim of the current study was to investigate the association of EEG activity in the delta, theta, alpha, and beta bands during different rest conditions with the cognitive status of OA, ranging from healthy to MCI (aMCI and naMCI). Since cognitive changes in the course from healthy aging to early dementia describe a continuum, the exact diagnostic classification of MCI is difficult (Petersen, 2004). This might become even more difficult with the advancing age of the sample. In order to tackle this uncertainty, we categorized OA into groups of different cognitive status, taking into consideration the level of evidence of cognitive impairments (see Methods) and using the recommendations for diagnosis of MCI in community-based samples (Petersen et al., 2018). This resulted in three groups: (1) cognitively healthy individuals (CHI) with strong evidence of no cognitive impairments, (2) possible MCI (pMCI) subjects with some evidence of cognitive impairments, and (3) MCI participants with strong evidence of cognitive impairments (Müller et al., 2020). The MCI group was further subdivided according to type of cognitive deficits in aMCI and naMCI. As the prevalence of MCI is positively correlated with age (Kryscio et al., 2006), only high agers (participants in their eighties) were included in the study to ensure a sufficient amount of MCI cases in the volunteer sample. Also, this was supposed to fill the previously identified gap for data from high-agers in the context of MCI research.

The main objective was to find out if the rsEEG of 80+-year-olds with MCI (pMCI, aMCI, and naMCI) differed significantly from healthy individuals of the same age. Therefore, differences between groups in mean absolute and mean relative power of the delta, theta, alpha, and beta bands were studied for EO, EC, and reactivity (EO–EC). It was expected that, similar to findings in younger samples of MCI and samples of patients with AD, MCI would have lower alpha and beta power and stronger delta and theta power during EC. In the EO condition, alpha power was expected to decrease in the MCI groups, while, for the other bands, no specific predictions could be made according to prior findings. Alpha reactivity was predicted to be smaller in the MCI groups, while no predictions were made for the other frequency bands.

## Methods

This study is part of the SENDA study (Sensor-based systems for early detection of dementia, registered in the German Clinical Trials Register under DRKS00013167), which was conducted at Chemnitz University of Technology, Germany. The detailed study protocol was published earlier by Müller et al. (2020). Only information relevant to the current research question will be described here.

### Participants

The SENDA study sample included 244 participants (123 males; age, 79–93 years; *M* = 82.5; *SD* = 2.5), which were recruited from January 2018 to March 2020. Study participation required walking ability, sufficient German language skills, residence in or around Chemnitz, Germany, and self-organized means of travel to and from the laboratory. Volunteers were excluded before testing if any of the following criteria applied: (1) acute psychological disorder; (2) diagnosis of any neurocognitive or neurological disorder; (3) past traumatic head injury; (4) substance abuse; (5) participation in other clinical studies; (6) a physician-directed ban from physical activities; (7) severe restrictions due to cardiovascular, pulmonary, or orthopedic diseases; (8) or failure to reach the minimum required score of 19 during screening with the Montreal Cognitive Assessment (MoCA, Nasreddine et al., 2005). Each participant signed a written informed consent, and all study proceedings were approved by the Ethics Committee of Chemnitz University of Technology, Germany, Faculty of Behavioral and Social Sciences (V232-17-KM-SENDA-07112017, approved on 19.12.2017). Each participant received 25 € compensation for his or her participation at three appointments. This included neuropsychological testing (part of first appointment) and EEG recordings (part of the second appointment).

The analysis for this article included 213 participants. Exclusion from analysis was due to (1) dropout from the study before all needed testing was completed (*n* = 17), (2) signs of severe depressive symptoms [Geriatric Depression Scale (Gauggel and Birkner, 1999) short version > 8, *n* = 9], (3) technical issues during the EEG recording, (*n* = 4), (4) and falling asleep during EEG recording (*n* = 1). Demographic characteristics are reported in Table 1. In addition, the participants reported their medication regimens. Due to the old age of the participants, many of them were following a medication regimen, which most often included medication for high blood pressure, thrombosis prophylaxis, cholesterol reduction, stomach acid reduction, and thyroid function. There were 15 participants taking medication, which might have influenced EEG activity, such as tricyclic antidepressants (*n* = 6), antipsychotics (*n* = 2), Parkinson medication (*n* = 2), anti-dementia medication (*n* = 2), and benzodiazepines (*n* = 5, prescribed for sporadic, not regular use, according to medication plans). These cases were distributed across all four groups (CHI: 3, pMCI: 4, naMCI: 5, and aMCI: 3). Conducting the following analysis without these cases did not result in any differences, and we, therefore, did not remove them from the sample.

**Table 1:** Characteristics of the total sample and groups according to cognitive status.

	**Total**		**CHI**		**pMCI**		**naMCI**		**aMCI**		
N (in %)	213 (100)		72 (34)		80 (38)		17 (8)		44 (21)		
m/f	109/104		32/40		43/37		12/5		22/22		
Age in years *M* (*SD*)	82.5	(2.4)	82.1	(2.4)	82.5	(2.1)	83.2	(3.1)	83.0	(2.7)	
Education in years *M* (*SD*)	14.0	(3.2)	14.4	(3.4)	14.0	(3.3)	14.3	(3.2)	13.3	(2.7)	
MoCA (0–30) *M (SD)*	25.6	(2.6)	27.8	(1.2)	25.8	(2.1)	22.8	(1.6)	22.8	(1.7)	^*^ ^a^
Handedness (-100–100) *M (SD)*	83.3	(38.2)	89.1	(24.4)	81.9	(41.6)	78.2	(39.5)	81.5	(42.6)	
GDS Score (0–15) *M* (*SD*)	2.8	(2.0)	2.6	(1.9)	2.6	(1.8)	3.6	(2.7)	3.4	(2.1)	
NAA Score (20–60) *M* (*SD*)	26.3	(3.4)	25.3	(2.7)	25.9	(3.3)	29.5	(4.5)	27.9	(3.0)	^*^ ^b^

*CHI, cognitively healthy individuals; pMCI, possible mild cognitive impairment; naMCI, non-amnestic MCI; aMCI, amnestic MCI; MoCA, Montreal Cognitive Assessment; GDS, Geriatric Depression Scale; NAA, Nürnberger-Alters-Alltagsaktivititäten-Skala (Nuremberg Gerontopsychological Rating Scale for Activities of Daily Living).*

* *p < 0.05.*

a *Post-hoc Dunn Bonferroni test showed: CHI > pMCI > naMCI = aMCI.*

b *Post-hoc Dunn Bonferroni test showed: CHI = pMCI < naMCI = aMCI*.

### Neuropsychological Testing and MCI Classification

All the participants went through an intensive neuropsychological test battery, which was carried out from trained testing staff at the University lab. This included the German version of the MoCA (Nasreddine et al., 2005) and the German version of the Consortium to Establish a Registry for Alzheimer's Disease Neuropsychological Test Battery (Morris et al., 1989; Memory Clinic Basel, 2005; CERAD-NP). The MoCA was used to measure global cognitive functioning and to screen for MCI. It is the second most utilized geriatric cognitive screening tool after the mini mental status examination but has superior sensitivity to mild cognitive impairments (Breton et al., 2019). The CERAD-NP examines the cognitive domains memory, language, executive functions, and visuo-construction. In addition, information about the level of education (overall years of education) and handedness [a laterality quotient according to Oldfield (1971)] was obtained. The participants completed additional questionnaires at home, which included, among others, the Nürnberger-Alters-Alltagsaktivititäten-Skala (NAA; Nuremberg Gerontopsychological Rating Scale for Activities of Daily Living; Oswald and Fleischmann, 1995) to measure basic and instrumental activities of daily living as well as the German short version of the Geriatric Depression Scale (GDS; Gauggel and Birkner, 1999) to screen for depressive symptoms. The GDS was used to exclude individuals from the analysis (GDS > 8) to prevent the inclusion of undetected cases of major depression and also as a covariate.

MCI classification was based on the recommendations of The National Institute on Aging and the Alzheimer's Association (Albert et al., 2011) and in accordance with the criteria proposed by Petersen et al. (2014). These criteria are also part of the Diagnostic and Statistical Manual of Mental Disorders (5th ed.; DSM-5; American Psychiatric Association, 2013) for the diagnosis of mild neurocognitive disorders. The criteria were: (1) self—or informant report of cognitive complaints, (2) impairments in at least one cognitive domain while taking into consideration age and education, (3) general independence in daily activities, and (4) no dementia. Cognitive complaints (criteria 1) were not included as a criterion of MCI here because there is no consensus on inclusion or operationalization (Mitchell, 2008). Subjective complaints also seem to be far less relevant for the prediction of dementia in community-based samples like ours compared with the participants in memory clinics (Snitz et al., 2018). In addition, we found subjective complaints to be very common in this age group. In a subgroup of our sample (*n* = 136), 65% of the participants reported memory complaints when asked to compare their memory performance 5 years prior.

Cognitive impairments (criteria 2) were determined according to performance in MoCA (one sum score) and CERAD-NP (nine separate test scores). The following CERAD-NP scores were used: verbal fluency (number of animals named in 1 min), Boston naming test (number of objects correctly identified), phonematic fluency (number of words named with letter “S” in 1 min), constructional praxis (number of correctly copied characteristics), word list learning (number of words correctly remembered in third trial), word list recall (savings score), word list recognition (discriminability score), constructional praxis recall (savings score), and trail making test (quotient B/A). We followed a two-step procedure that is recommended for diagnosis of MCI in the general population, which states that, first, a screening should be used, and, in case of abnormal findings, in-depth cognitive testing should follow (Petersen et al., 2018). A MoCA score below 26 points and at least one CERAD-NP performance below 1.5 standard deviations of the normative mean (taking into consideration age, sex, and education level) resulted in the classification of mild cognitive impairment (MCI). Correspondingly, the participants were classified as being healthy (CHI) if they scored 26 or more points on the MoCA and also within the normative range (no score below −1.5 SD) in all of the CERAD-NP scores. Out of the participants classified as MCI, amnestic cases (aMCI) were distinguished by deficits in at least one of the memory tests (word list learning, word list recall, word list recognition, and constructional praxis recall). Accordingly, non-amnestic cases (naMCI) presented with deficits only in the other non-memory tests. Due to the application of the two-step process, an additional class was defined for the participants who showed cognitive impairments only according to one of the two tests. They were categorized as possibly having MCI (pMCI). This group either included the participants who had deficits in one specific domain of the CERAD-NP, but, overall, cognitive functioning was normal according to MoCA or the participants that had no strong impairment in any single domain, but small deficits in different domains added up to a low MoCA score (<26). Although this group would be considered as non-MCI according to Petersen et al. (2018) as these individuals neither showed abnormal scores in the screening (MoCA > 25) nor in-depth clinical testing after abnormal testing revealed any cognitive impairments, we opted to separately analyze this group to have high discriminatory power between CHI and MCI.

General independence (criteria 3) was presumed for all the participants because we only included community-dwelling volunteers in this study. This was further confirmed by the NAA scores, which were below 39 for all individuals and fell within a normal range for this age group (Oswald and Fleischmann, 1995). No dementia (criteria 4) was also ensured due to the exclusion criteria described before.

### EEG Recordings

The actiCHamp system (Brain Products GmbH, Gilching, Germany) was used to record 32 active EEG electrodes positioned according to the international 10–20 system (Fp1, Fp2, F7, F3, Fz, F4, F8, FC5, FC3, FC1, FC2, FC4, FC6, T7, C3, Cz, C4, T8, CP5, CP3, CP1, CP2, CP4, CP6, P7, P3, Pz, P4, P8, O1, Oz, and O2). The setup included a forehead ground electrode at Fpz and an online reference electrode at Fz. All data were acquired with a 500 Hz sampling rate and 24-bit resolution. The electrode-skin impedance was kept below 25 kΩ.

**Figure 1:**
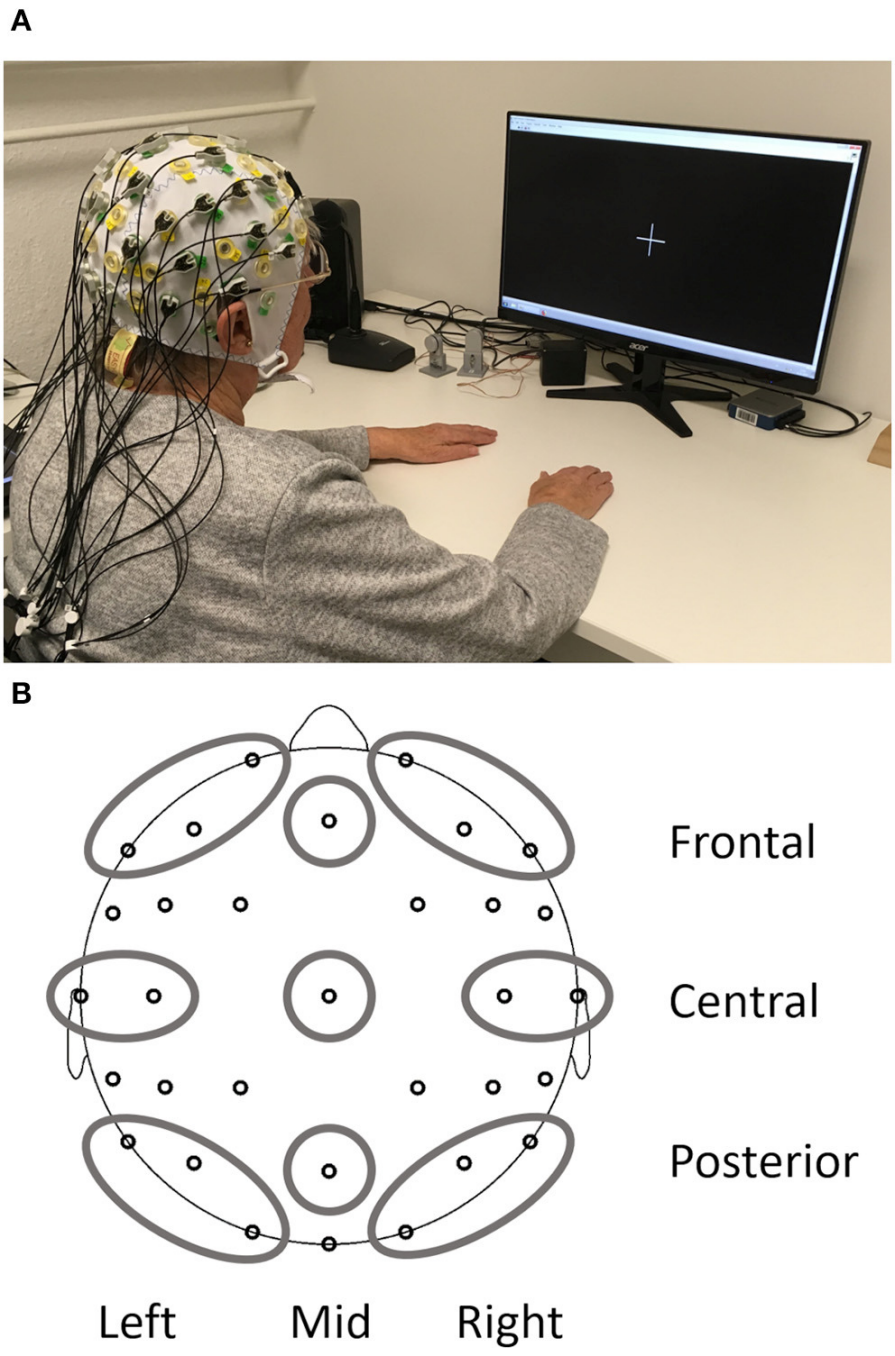
Setup of the resting state EEG measurements **(A)** and depiction of the nine regions of interest obtained from the EEG **(B)**.

The EEG recording during rest only made up a small part of the complete testing on the day and always took place after gait analysis and prior to fine motor testing. Rest periods were offered during the whole procedure, and all the participants had received a short break prior to EEG recording. EEG measurements took place in an electrically shielded and darkened room. To minimize EEG artifacts and distractions for the subject, all instructions were given from an adjacent room *via* a microphone and a monitor. The participants sat relaxed, with their backs leaned against the back rest and both hands rested comfortably on the table in front of them (see Figure 1A for a photo of the complete setup). They looked at a white fixation cross at the center of a black screen for 4 min (condition EO) and, afterwards, closed their eyes for 2 min (condition EC). The level of consciousness the subject was monitored to annotate changes and other artifacts in the EEG protocol.

### Preprocessing of EEG Data

BrainVision Analyzer 2.2 (Brain Products GmbH, Gilching, Germany) was used for all preprocessing steps. Data were filtered (phase shift-free Butterworth infinite impulse response filter, 1–70 Hz, slope 48 dB/Hz), notch filtered (50 Hz), and down sampled from 500 to 256 Hz. In addition, blink artifacts in the rest condition EO were removed *via* Independent Component Analysis (Jung et al., 1998) with Fp1 as the reference channel for vertical eye movements. Continuous EEG data were then common average re-referenced and segmented into 2-s epochs for an automatic artifact rejection. Epochs were rejected from further analysis if at least one channel included voltage steps >25 μV/ms or if the difference between minimal and maximal absolute voltage recorded exceeded 200 μV in any 200 ms interval.

At each electrode absolute power (in μV^2^) and relative power (in %, relative to the total power of the spectrum 1–24 Hz) was calculated with a Fast Fourier Transform algorithm for each 2s epoch resulting in 0.5 Hz resolution. A Hanning window (length 10%) and variance correction were applied to correct for spectral leakage. Mean absolute power and mean relative power were obtained by averaging 15 artifact-free segments for 30 s after the start of the condition. One participant did not have enough artifact free segments for the EO and another participant for the EC condition. Therefore, EO and EC analyses were carried out with *N* = 212 and the reactivity analysis with *N* = 211. Frequency bands included delta (1–3.5 Hz), theta (4–7.5 Hz), alpha (8–13 Hz), and beta (13.5–24 Hz). All data were log-transformed (base 10) to obtain normal distribution and variance homogeneity before calculation of regions of interest (ROI) based on Barry and De Blasio (2017). The combination of three sagittal planes (left, mid, and right) and three coronal planes (frontal, central, and posterior) resulted in nine different ROIs (Figure 1B): left frontal (Fp1, F3, and F7), mid frontal (Fz), right frontal (Fp2, F4, and F8), left central (T7 and C3), mid central (Cz), right central (T8 and C4), left posterior (P7, P3, and O1), mid posterior (Pz), and right posterior (P8, P4, and O2). Reactivity for absolute and relative power was calculated separately for each frequency band as the difference between EO and EC (log power EO- log power EC) for each ROI.

In addition, from the same spectrum (relative power, EC condition, 30 s), we also obtained the individual alpha frequency (IAF) for each person. All electrodes of the posterior region (P7, P3, O1, Pz, P8, P4, and O2) were averaged, and the frequency of the maximum value in the alpha band was extracted with the MinMax Marker Solution (BrainVision Analyzer 2.2). Six participants were not included in this analysis because they did not show clear peaks in the alpha range. This was indicated by the values of the detected peak being less than 1.96 standard deviations above the mean value of the alpha range. Visual inspection of the cases indicated either absence of a peak or a peak in the theta range.

### Statistical Analysis

IBM SPSS Statistics Version 27 (IBM Corp., Armonk, NY, USA) was used for all statistical analysis. *P-*values < 0.05 were regarded as significant and *p*-values < 0.10 as a trend unless they had to be adjusted for multiple testing. Effect sizes were reported as partial eta squares (η${}_{\text{p}}^{2}$). As variables were not normally distributed, Kruskal–Wallis tests were used to test if covariates age, education, and depressive symptoms differed between groups. A chi-square test was used to test if sex and group distributions were independent. No significant differences between groups emerged for any of the covariates, which means that potential effects of cognitive status on EEG parameters should not be due to sex, age, and education confounding with the group classification.

First, absolute power data were pre-analyzed in order to check if reactivity was still preserved in the sample of high-agers. For this purpose, a 2 × 3 × 3 × 4 mixed-design ANOVA was carried out with the three within-subject factors rest condition (EO, EC), sagittal (left, mid, and right) and coronal (frontal, central, and posterior) as well as one between-subject factor group (CHI, pMCI, naMCI, and aMCI), and the main effect of rest condition was reported for each frequency band.

All the following analyses were run with sex as covariate. Age and education in years were not included as covariates because there was no significant relationship with any of the EEG parameters, and their inclusion did not improve variance explanation. One-way analysis of covariance (ANCOVA) was used to test for differences in IAF between groups. Next, six 3 × 3 × 4 mixed-design ANCOVAs were carried out with the dependent variables (1) absolute EC power, (2) absolute EC power, (3) absolute power reactivity, (4) relative EO power, (5) relative EO power, and (6) relative power reactivity, respectively. Each ANCOVA included two within-subject factors sagittal (left, mid, and right) and coronal (frontal, central, and posterior) as well as one between-subject factor group (CHI, pMCI, naMCI, and aMCI) to find differences between groups and topography. Greenhouse-Geisser adjustments were reported whenever sphericity assumptions were violated. To control for multiple testing within each frequency band (three tests for absolute power and three tests for relative power), the Bonferroni adjusted alpha level of 0.017 was used. Last, the directions of the significant main and interaction effects from the 3 × 3 × 4 ANCOVAs were determined *via* contrast analysis to describe the topography in more detail. For the coronal factor, two contrasts were used: comparing frontal with posterior (F – P) and comparing the mean of frontal and posterior against the central ROI (F/P – C). Similarly, two contrasts were included for the sagittal factor: comparing left with right (L – R) and comparing the mean of left and right with the mid ROI (L/R – M). Again, Bonferroni adjusted alpha levels were used to control for testing multiple contrasts within one effect (main effects: 0.025 interaction effect: 0.0125). Only significant effects are reported in the text unless stated otherwise.

## Results

### Reactivity

Results from the 2 × 3 × 3 × 4 mixed-design ANOVA indicated a significant reduction in absolute power from EC to EO across the whole sample in delta [*F*_(1, 207)_ = 30, *p* < 0.001, η_p_^2^ = 0.13], theta [*F*_(1, 207)_ = 144.4, *p* < 0.001, η_p_^2^ = 0.41], alpha [*F*_(1, 207)_ = 275.3, *p* < 0.001, η_p_^2^ = 0.57], and beta bands [*F*_(1, 207)_ = 6.6, *p* = 0.01, η_p_^2^ = 0.03].

### Cognitive Status

Classification of participants in the four groups (CHI, pMCI, aMCI, and naMCI) according to the introduced criteria resulted in 72 CHI, 80 pMCI, 17 naMCI, and 44 aMCI cases (Table 1). The four groups differed significantly according to problems with daily activities measured with the NAA (CHI = pMCI < aMCI = naMCI). The IAF was fastest in the healthy group (*M* = 9.3 Hz, *SD* = 1.1) compared with the groups with cognitive impairments (pMCI: *M* = 9.0 Hz, *SD* = 0.8, naMCI: *M* = 9.1 Hz, *SD* = 0.8, aMCI: *M* = 9, *SD* = 0.8). These differences were not significant [*F*_(3, 206)_ = 1.6, *p* = 0.19, η_p_^2^ = 0.02].

**Figure 2:**
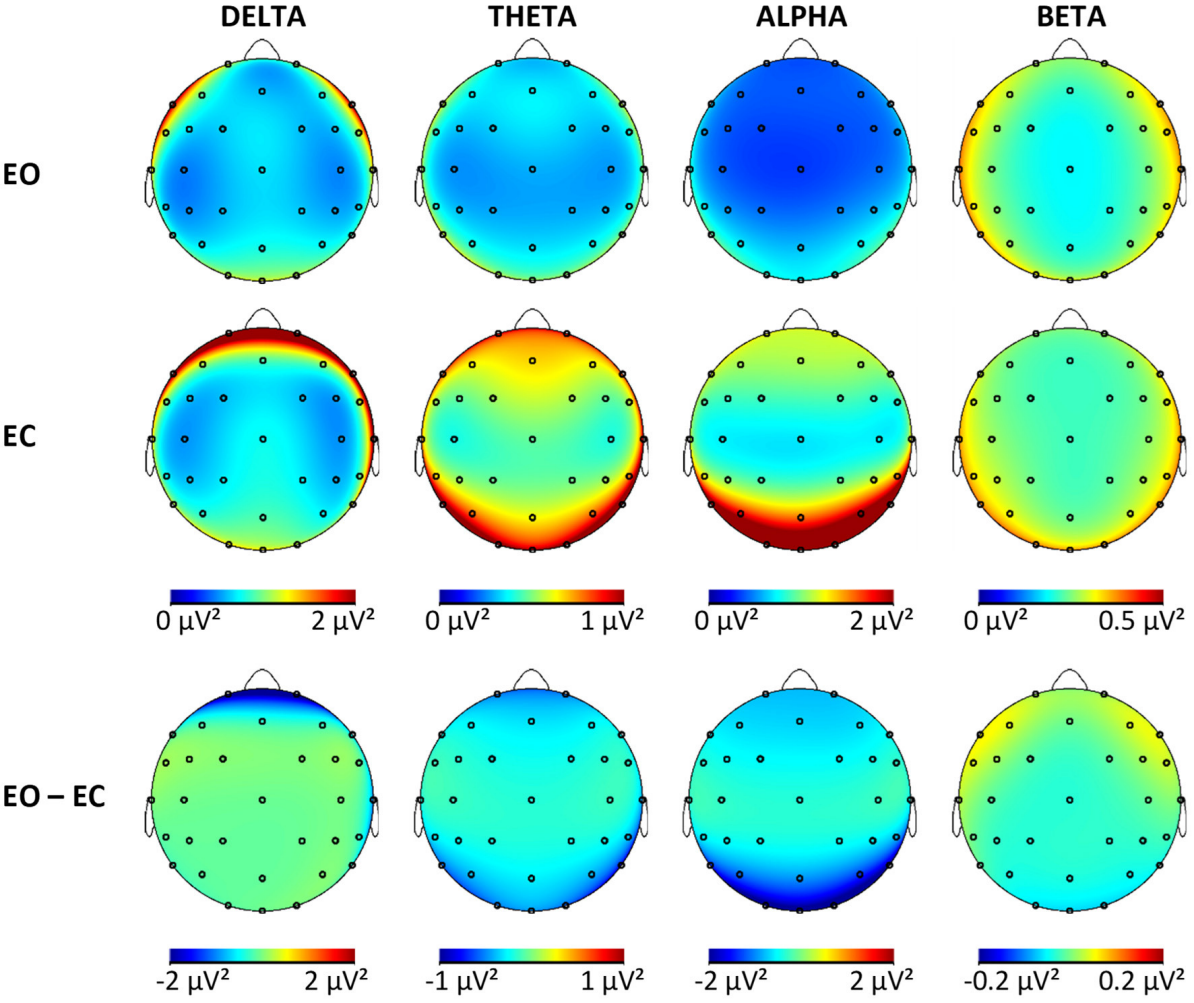
Brain maps showing the mean absolute power in μV^2^ for all frequency bands in both conditions and the difference maps. EO, eyes open; EC, eyes closed.

Tables with log-transformed absolute power values for each frequency band, group, and ROI are available in the Supplementary Material. Results of the mixed ANCOVA for each frequency band for the outcome variables (power EC, power EC, and reactivity) revealed no significant group effects or interactions involving the factor group for neither absolute nor relative power analysis. The p-values for these nonsignificant effects ranged from *p* = 0.05 to *p* = 0.90 (with effect sizes between η_p_^2^ = 0.00 and η_p_^2^ = 0.04) for absolute power and *p* = 0.02 to *p* = 0.98 (with effect sizes between η_p_^2^ = 0.01 and η_p_^2^ = 0.04) for relative power. In this sample, the rsEEG activity in the four frequency bands did not differ significantly according to the cognitive status of the participants when using absolute or relative power values. As no differences between groups were established, all the participants were pooled together to obtain brain maps from the non-transformed absolute power values (Figure 2) for each frequency band and condition for this sample of OA to illustrate the topographies. The maps for each group separately are available in the Supplementary Material. In the whole sample, the effects were significant for the sagittal factor, the coronal factor, and the interaction between sagittal and coronal for all frequency bands and absolute power outcomes. The topographical effects will be looked at in more detail in the following sections only for absolute power. Relative power values are especially useful to control for person-specific confounding variables, which are less relevant to within-subject effects. In addition, differences in relative power are less clear to interpret because they can be caused by changes in the studied frequency band or changes in any of the other bands used in normalization. The results from the topographical analysis of relative power are available in the Supplementary Material.

### Topography During EC

The complete results of the contrast analysis can be seen in Table 2. For all frequency, bands activity was significantly smaller at the midline compared to hemispheres (L/R > M). There was no effect of lateralization in any of the frequency bands (L = R). Both alpha and beta were dominant in the posterior regions (F < P), while delta band was dominant in the frontal region (F > P). For the delta, theta, and alpha bands, central activity was less pronounced compared with the mean activity from frontal and posterior (F/P > C). In the delta band, the difference between midline and hemispheres was more pronounced frontally compared with the posterior regions (L/R > M × F > P). For all other bands, this was reversed with stronger differences between midline compared with L/R in the posterior regions instead of frontal regions (L/R > M × F < P). Although no global effect of lateralization was obtained in the alpha band, there was more pronounced activity in the right hemisphere of the posterior region (L < R × F < P). The smallest power values for theta and delta were obtained from the mid-central regions (L/R > M × F/P > C).

**Table 2:** Results of the contrast analysis in each frequency band for absolute power (log-transformed) at rest with eyes closed.

	**Delta**			**Theta**			**Alpha**			**Beta**		
	***F***	***p***	***η_*p*_^2^***	***F***	***p***	***η_*p*_^2^***	***F***	***p***	***η_*p*_^2^***	***F***	***p***	***η_*p*_^2^***
**Main Effects (adj**. ***α*****-level=0.025)**												
L > R	4.1	0.04	0.02	1.9	0.18	0.01	1.1	0.129	0.01	0.0	0.89	0.00
L/R > M	266.5	** <0.001**	0.56	264.4	** <0.001**	0.56	387.4	** <0.001**	0.65	141.5	** <0.001**	0.41
F > P	130.5	** <0.001**	0.39	1.1	0.29	0.01	236.4	**<**0.001	0.53	26.4	**<**0.001	0.11
F/P > C	135.7	** <0.001**	0.40	165.3	** <0.001**	0.44	115.0	** <0.001**	0.36	3.1	0.08	0.02
**Interactions (adj**. ***α*****-level=0.0125)**												
L > R x F > P	1.4	0.23	0.01	0.4	0.51	0.00	11.9	**0.001**	0.05	3.1	0.08	0.02
L > R x F/P > C	2.2	0.14	0.01	0.2	0.62	0.00	2.6	0.11	0.01	0.8	3.8	0.00
L/R > M x F > P	86.7	** <0.001**	0.30	4.7	0.03	0.02	81.1	**<**0.001	0.28	15.2	**<**0.001	0.07
L/R > M x F/P > C	15.9	** <0.001**	0.07	8.1	**0.005**	0.04	1.3	0.26	0.01	1.2	0.28	0.01

*All test statistics are with (1, 207) degrees of freedom. Underlined effects are reversed in direction (i.e., the reversed effect from L > R × F > P is L < R × F > P). Changing the direction of both directional indicators within a single effect is equivalent (i.e., L > R × F > P is the same as L < R × F < P). L, left; R, right; M, midline; F, frontal; P, posterior; C, central. Significant results are printed in bold*.

### Topography During EO

The topography during the EO was very similar to EC topography (Tables 2, 3). The only differences pertained to lateralization, where theta and alpha both showed greater power in the left compared with the right hemisphere (L > R) and no differences in lateralization between frontal and posterior regions.

**Table 3:** Results of the contrast analysis in each frequency band for absolute power (log-transformed) at rest with eyes open.

	**Delta**			**Theta**			**Alpha**			**Beta**		
	***F***	***p***	***η_*p*_^2^***	***F***	***p***	***η_*p*_^2^***	***F***	***p***	***η_*p*_^2^***	***F***	***p***	***η_*p*_^2^***
**Main Effects (adj**. ***α*****-level=0.025)**												
L > R	3.6	0.06	0.02	6.1	**0.02**	0.03	11.4	**0.001**	0.05	1.7	0.18	0.01
L/R > M	158.6	** <0.001**	0.43	173.7	** <0.001**	0.46	408.9	** <0.001**	0.66	143.4	** <0.001**	0.41
F > P	39.0	** <0.001**	0.16	2.9	0.09	0.01	106.1	**<**0.001	0.34			
F/P > C	50.7	** <0.001**	0.20	86.4	** <0.001**	0.29	12.0	**0.001**	0.06			
**Interactions (adj**. ***α*****-level=0.0125)**												
L > R x F > P	0.0	0.87	0.00	2.6	0.11	0.01	0.0	0.95	0.00	6.7	0.01	0.03
L > R x F/P > C	1.4	0.23	0.01	0.1	0.80	0.00	0.5	0.47	0.00	0.5	0.49	0.00
L/R > M x F > P	24.1	** <0.001**	0.10	16.5	**<**0.001	0.08	14.8	**<**0.001	0.07	0.6	0.45	0.00
L/R > M x F/P > C	1.4	0.12	0.01	0.1	0.72	0.00	45.2	**<**0.001	0.18	7.4	0.01	0.03

*All test statistics are with (1,207) degrees of freedom. Underlined effects are reversed in direction (i.e., the reversed effect from L > R × F> P is L < R × F > P). Changing the direction of both directional indicators within a single effect is equivalent (i.e., L > R × F > P is the same as L < R × F < P). L, left; R, right; M, midline; F, frontal; P, posterior; C, central. Significant results are printed in bold*.

### Topography of Reactivity

Topographical differences in reactivity were apparent in the descriptive reactivity data (Supplementary Material) and were confirmed by the contrast analysis (Table 4). When interpreting the direction of effects, the sign of the reactivity values must be considered. When comparing two negative values, the smaller value is the more negative value and, therefore, indicates the larger change from EC to EO.

**Table 4:** Results of the contrast analysis in each frequency band for reactivity (difference of log-transformed absolute power).

	**Delta**			**Theta**			**Alpha**			**Beta**		
	***F***	***p***	**η_p_^2^**	***F***	***p***	**η_p_^2^**	***F***	***p***	**η_p_^2^**	***F***	***p***	**η_p_^2^**
**Main Effects (adj**. ***α*****-level=0.025)**												
L > R							21.8	** <0.001**	0.10	1.3	0.25	0.01
L/R > M							9.1	**0.003**	0.04	18.7	** <0.001**	0.08
F > P	18.0	**<**0.001	0.08	0.1	0.76	0.00	66.2	** <0.001**	0.24	29.3	** <0.001**	0.12
F/P > C	39.7	**<**0.001	0.13	25.9	**<**0.001	0.11	86.6	**<**0.001	0.30	0.5	0.48	0.00
**Interactions (adj**. ***α*****-level=0.0125)**												
L > R x F > P	0.6	0.43	0.00	4.5	0.04	0.02	8.5	0.004	0.04	2.2	0.14	0.01
L > R x F/P > C	0.1	0.76	0.00	0.1	0.80	0.00	1.0	0.33	0.01	0.1	0.77	0.00
L/R > M x F > P	11.5	0.001	0.05	2.83	0.09	0.00	30.6	** <0.001**	0.13	7.9	**0.01**	0.04
L/R > M x F/P > C	8.9	0.003	0.04	8.0	0.01	0.04	28.3	**<**0.001	0.12	5.5	0.02	0.03

*All test statistics are with (1,206) degrees of freedom. Underlined effects are reversed in direction (i.e., the reversed effect from L > R × F > P is L < R × F > P). Changing the direction of both directional indicators within a single effect is equivalent (i.e., L > R × F > P is the same as L < R × F < P). L, left; R, right; M, midline; F, frontal; P, posterior; C, central. Significant results are printed in bold*.

For the delta band, the pattern of reactivity resembled that of the EC condition, which means that the greatest changes from EC to EO were present in the areas with the most delta activity during EC [F < P; F/P < C; L/R < M × F > P; L/R < M × F/P > C]. For the theta, band reactivity was less pronounced in the central regions (F/P < C), specifically the left and right hemispheres (L/R > M × F/P < C), which were also the regions with less theta activity in EO and EC. In the alpha band, once again, reactivity was more pronounced in the right compared with the left hemisphere (L > R), which explained the change from a right hemispheric bias during EC to a significant left hemispheric bias during EO. Further considerations of interactions actually showed that this was only the case in the posterior but not the frontal region (L > R × F < P). The change from EC to EO in alpha power was greater in the midline compared with hemispheres (L/R > M), especially so in the frontal regions (L/R > M × F > P). Reactivity was strongest in the posterior region and least pronounced in the central regions (F > P, F/P < C), which reproduces the pattern of alpha activity during EC. In the beta band, reactivity was more pronounced in the midline compared with the hemisphere (L/R > M) and in the posterior compared with frontal regions (F > P). This is related to the fact that beta activity in the hemispheres is increasing in the left and right frontal regions while it is decreasing with the opening of eyes in the other regions (L/R > M × F > P). This focal frontoparietal activity with opening the eyes can also be seen in Figure 2 (last column).

## Discussion

In this study, the synchronized activity at rest while eyes are open and closed in the classical broad bands delta, theta, alpha, and beta was compared between cognitively healthy OA and individuals with MCI of the same age. The sample included OA, 80 years or older, which are often not enough represented in studies on early detection of dementia. Groups were compared with respect to mean absolute power, relative power, and reactivity to eyes opening separately in each band. No significant differences between any of the groups of different cognitive status (CHI, pMCI, naMCI, and aMCI) were detected. Overall, specific topographical patterns were present, which will be compared with results from other age groups later. In addition, EEG reactivity was also present in each of the four frequency bands with overall greater power during EC compared with EO and a few focal increases in the beta band. The topography of reactivity for the most part related to the topography found in the EC condition.

No significant differences between any of the groups of different cognitive status were found in IAF or resting state power in EC, and, therefore, it can be concluded that the absolute and relative power distributions were similar in each of the four groups (CHI, pMCI, naMCI, and aMCI) for this condition. Thus, the hypotheses that MCI is characterized by lower alpha and beta power as well as stronger delta and theta power during EC could not be confirmed in our sample. This is not in complete agreement with prior findings of changes in the rsEEG in patients with MCI. For the rest with EC, it was shown that alpha and beta powers were reduced and theta and delta powers were either elevated or reduced in MCI compared with healthy OA (Koenig et al., 2005; Babiloni et al., 2006b, 2010; Kwak, 2006; Ya et al., 2015). In fact, when specifying former studies, each study only showed some of the listed changes, but the overlap between results was often not great even though similar parameters were studied.

One might assume that the lack of significant differences between MCI and healthy participants in our study was caused by an unsuitable resting state measurement protocol. This seems to be rejectable as the protocol was very comparable to the ones used in other MCI and dementia studies (e.g., Alexander et al., 2006; van der Hiele et al., 2007b; Gaál et al., 2010; Toth et al., 2014).

One major difference between the current findings and that of other studies was the overall older age (mean, 82.5 years) of the participants. The average age of most study samples was ~10–20 years below that of the present sample [e.g., 62 years (Koenig et al., 2005), 68 years (Barry and De Blasio, 2017), and 72 years (Babiloni et al., 2006b)]. In addition, the number of rsEEG studies in this age group is very limited, which means that there is limited knowledge of the typical rsEEG in MCI, but it is also unclear how the rsEEG activity of healthy high-agers looks. Some aging-related changes in the rsEEG, like the reduction in alpha power, are probably similar in the aging process and the neuropathological process of dementia (Rossini et al., 2007), and, therefore, it might be harder to differentiate between healthy but far advanced aging and early neuropathological changes. Postmortem studies also showed that dementia pathology, such as neuritic plaques, diffuse plaques, and neurofibrillary tangles can be found in healthy OA without signs of dementia or MCI during their lifetime (Bennett et al., 2006). In general, the overlap in neuropathology between healthy and individuals with dementia seems to increase with age (Richard et al., 2012). Taken all together, this suggests that the cognitive status of high-agers as determined by neuropsychological testing might not necessarily represent the underlying neurophysiological state.

For EEG measurements, it must also be considered that aging can cause anatomical changes that can dampen the measurable EEG signal. It has been shown that cortical thinning with aging results in smaller measurable EEG amplitudes and that power differences between different age groups can be explained by including cortical thickness into the analysis (Provencher et al., 2016). As a consequence, it might be statistically problematic to detect differences if the baseline level of power is very low. On average, this is not the case in the current sample. The power values at rest with EO in the present data set are comparable with values found in a prior study (Hübner et al., 2018) with younger OA (67–83 years).

The different groups of cognitive status were also compared with regard to resting state power while EO and reactivity (change from EC to EO). Although it had been shown before that EO conditions might be better suitable to detect EEG changes in MCI (McBride et al., 2014), this was not replicated here. The present results indicated no differences in resting state power with EO or reactivity according to cognitive status in any of the frequency bands. Thus, the hypothesis that MCI is characterized by reductions in alpha power during EO and reduced reactivity in the alpha band was not confirmed. In addition, for the first time, analysis of reactivity was not restricted to the alpha band and included also delta, theta, and beta bands. Group comparisons showed that reactivity in the other bands was also not related to cognitive status.

In addition, we studied the topography and reactivity of each frequency band without taking into consideration the cognitive status of the participants to generate knowledge about the rsEEG in a group of non-demented high-agers. The topography of the slower bands (delta and theta) was described with maximal power at the vertex in both rest conditions in healthy OA in prior studies (Barry and De Blasio, 2017). This topography was not replicated here, as delta power showed frontal dominance with the smallest power at the vertex. Theta power was also smallest in the central regions. It is unclear why these differences arise and if a small sample size of prior studies, EEG setup or artifacts could be the cause of this. As this pattern was especially pronounced during EC condition, which typically shows very little frontal artifacts such as blinking, this should not be the reason. Other studies with young participants actually reported a very similar pattern with prefrontal dominance of delta power (Barry et al., 2007; Chen et al., 2008).

For the alpha band, topography was similar and, as expected, showed strongest alpha power in the posterior ROI and smallest power values centrally. A right hemisphere bias was present in the alpha band during EC conditions and a left hemisphere bias in the alpha and theta bands during EO, while, for all other bands and conditions, no hemispherical differences were found. In comparison, younger adults showed a right hemisphere bias across all frequency bands during rest (EO and EC), which is assumed to arise from the dominance of the left hemisphere in right-handed participants (Simon-Dack et al., 2013; Barry and De Blasio, 2017). This difference between our sample and results from younger OA confirms many findings of age-related neural dedifferentiation (Koen and Rugg, 2019).

The changes in band power due to eyes opening, in general, resembled what has been shown in younger adults. Reactivity was present in all bands and showed the typical pattern of overall decreased power in all bands, and only focal frontal increases in the beta band in EO (Barry and De Blasio, 2017). Even in high-agers, reactivity is maintained in all frequency bands, showing intact regulation of arousal and vigilance in the different resting state conditions. The exact topographical pattern for delta, theta, and alpha bands related to the observed EC pattern in each band, meaning the difference EO – EC was the strongest in ROIs that showed the most activity during EC (delta: frontal, theta: frontal and posterior, alpha: posterior).

### Limitations

Some limitations of this study must be considered. First, all the participants were volunteers, without symptoms of dementia and no need to live in a nursing home. These constraints resulted in the sample having a bias toward comparatively healthy and well-educated individuals. Education could be an influencing factor, because it is known as a proxy of a cognitive reserve, and it can impact the relationship between brain changes and performance measured in neuropsychological testing (Liu et al., 2013). This should not influence the present results because the groups did not differ in their levels of education.

In addition, one might assume that the MCI cases found in this sample were mostly very mild and far from the progression to dementia. However, the range of MoCA scores (19–25) and the deficits found in CERAD-NP scores (<1.5 SD below age specific norms) for the MCI groups indicated that this is not the case. Although the norms of neuropsychological test batteries like the CERAD-NP can be very strict when used for individuals older than 75 years (Luck et al., 2018), this issue was resolved by using a two-step classification system to evaluate the cognitive status of the participants. This included the neuropsychological test battery (CERAD-NP) with age- and sex-corrected norm values and the MoCA. This screening tool is known to detect MCI well-compared with others like the Mini Mental Status Examination, which suffers from ceiling effects in populations with mild impairments (Larner, 2012; Breton et al., 2019). Standardized classification criteria according to recommendations of the neurotic National Institute on Aging and the Alzheimer's Association (Albert et al., 2011) were employed. This procedure is certainly comparable to the standard clinical procedure, which includes first a screening and then more extensive neuropsychological testing. In addition, this recruitment procedure was chosen to obtain a sample of OA with no or only mild cognitive deficits, as we were especially interested in those early preclinical stages of dementia. Other studies often used MCI samples that arose from memory clinics, where probably, individuals applied with complaints, indicating further progressive cognitive decline. Conversely, the present sample allowed to study the process of cognitive decline even earlier.

The prevalence of MCI obtained from this strategy was 29%, which is slightly higher than the incidence rate for community samples calculated in a recent meta-analysis (Hu et al., 2017). Considering the age of the sample, this prevalence seems well-fitting and supports the validity of the classification strategy used. In addition, the distribution from naMCI and aMCI matches with prior findings that aMCI is the most common type of MCI (Petersen et al., 2010). Unfortunately, a relatively large part of the sample was classified as pMCI, indicating the high rate of diagnostic uncertainty often apparent in the diagnosis of preclinical and early dementia (Dubois et al., 2016).

The present study only focused on a selection of EEG parameters that can be obtained from Fast Fourier transform (spectral analysis). This was done because such parameters have been shown before to differentiate between healthy and persons with mild impairment (Koenig et al., 2005; Babiloni et al., 2006b, 2010; Kwak, 2006; Ya et al., 2015). They were now applied to a high-ager sample to study their usefulness in terms of early detection of dementia in such age groups. It is possible that early changes in resting state networks are better found with other or more advanced analysis methods. For example, measures of complexity (i.e., frequency or time domain entropy) or functional connectivity (i.e., coherence, phase lag index, and synchronization likelihood, and others) are able to extract different information from the signals of resting state networks than absolute and relative power can (Babiloni et al., 2019). Signal complexity seems to be reduced in MCI compared with healthy OA, although there are only few studies, including MCI, in addition to AD cases (Sun et al., 2020). Functional connectivity in MCI has been reported both as increased or decreased compared with healthy OA (Lejko et al., 2020). This might be due to pathophysiological as well as compensational processes present in MCI (Lejko et al., 2020). Future studies should use these advanced measures in the oldest-old samples to clarify if they can add findings that spectral analysis was not able to disentangle.

### Conclusion and Outlook

In this study, the rsEEG during EC and EO conditions of OA with and without cognitive impairments was studied. MCI was not related to detectable changes in EEG power during rest, neither for EC nor EO, compared with healthy individuals. Reactivity in any frequency band was also not different between groups of different cognitive status. With this sample of individuals in their 80's, it was challenging to differentiate between cognitive deficits caused by aging processes and actual pathological changes, indicating MCI. However, by including only the participants of very old age, it was possible to generate rsEEG data for an understudied age sample, which can help to establish normative data and is maybe better transferable to the clinical context, where the majority of individuals being diagnosed with MCI and dementia is rather old.

The present study results are strictly cross-sectional, and, therefore, no statements on the trajectory of neuropsychological performance and electroencephalographic parameters can be made. All the participants were part of a longitudinal study at the Chemnitz University of Technology, Germany (SENDA, sensor-based systems for early detection of dementia), and measurements were repeated up to three times in intervals of 8 months. In the future, additional data analysis will be carried out. This will have two main advantages: (1) the validity of the MCI diagnosis can be increased by including neuropsychological data of more than one time point (Albert et al., 2011) and (2) the predictive value of EEG parameters for the further cognitive decline can be studied. So far, the accuracy obtained from such studies is not high enough for clinical applications but they are more promising than cross-sectional comparisons (Yang et al., 2019).

## Data Availability Statement

The raw data supporting the conclusions of this article will be made available by the authors, without undue reservation. Requests to access the datasets should be directed to Claudia Voelcker-Rehage, ( claudia.voelcker-rehage@uni-muenster.de).

## Ethics Statement

The studies involving human participants were reviewed and approved by Ethics Committee of the Chemnitz University of Technology, Faculty of Behavioral and Social Sciences (number V-232-17-KM-SENDA-07112017). The patients/participants provided their written informed consent to participate in this study.

## Author Contributions

SF: investigation, data curation, formal analysis, writing—original draft, and visualization. DK: conceptualization, writing review and editing, and supervision. KM: project administration, investigation, data curation, and writing review and editing. CV-R: conceptualization, funding acquisition, resources, writing review and editing, and supervision. All authors contributed to the article and approved the submitted version.

## Conflict of Interest

The authors declare that the research was conducted in the absence of any commercial or financial relationships that could be construed as a potential conflict of interest.

## Publisher's Note

All claims expressed in this article are solely those of the authors and do not necessarily represent those of their affiliated organizations, or those of the publisher, the editors and the reviewers. Any product that may be evaluated in this article, or claim that may be made by its manufacturer, is not guaranteed or endorsed by the publisher.
