# Supplementary material for: Characteristics of Resting State EEG Power in 80+-Year-Olds of Different Cognitive Status
Source: Front Aging Neurosci. 2021 Aug 11;13:675689. doi: 10.3389/fnagi.2021.675689 (PMC8387136; doi:10.3389/fnagi.2021.675689)
Supplement: Supplementary file 1 [file Data_Sheet_1.pdf]

Supplementary Material

Supplementary Table 1. Log-transformed EC and EO rest absolute power in delta band (1 – 3.5 Hz) as M (SD) for each ROI and group.

		CHI			pMCI			naMCI			aMCI		
		Left	Mid	Right	Left	Mid	Right	Left	Mid	Right	Left	Mid	Right
EC	Frontal	0.74 (0.31)	0.24 (0.28)	0.72 (0.32)	0.71 (0.33)	0.25 (0.27)	0.73 (0.36)	0.66 (0.33)	0.27 (0.34)	0.65 (0.35)	0.79 (0.34)	0.28 (0.29)	0.76 (0.37)
	Central	0.33 (0.26)	0.17 (0.31)	0.31 (0.25)	0.36 (0.28)	0.16 (0.31)	0.36 (0.37)	0.24 (0.28)	0.15 (0.28)	0.24 (0.28)	0.41 (0.33)	0.25 (0.34)	0.34 (0.35)
	Posterior	0.39 (0.25)	0.25 (0.27)	0.37 (0.25)	0.42 (0.25)	0.31 (0.29)	0.41 (0.24)	0.38 (0.28)	0.30 (0.29)	0.37 (0.26)	0.46 (0.29)	0.33 (0.30)	0.46 (0.32)
EO	Frontal	0.45 (0.29)	0.15 (0.31)	0.43 (0.25)	0.47 (0.30)	0.17 (0.27)	0.48 (0.26)	0.50 (0.32)	0.18 (0.17)	0.52 (0.33)	0.61 (0.41)	0.22 (0.29)	0.58 (0.37)
	Central	0.27 (0.24)	0.13 (0.29)	0.28 (0.24)	0.31 (0.26)	0.11 (0.28)	0.29 (0.27)	0.29 (0.26)	0.08 (0.20)	0.24 (0.26)	0.40 (0.38)	0.25 (0.37)	0.37 (0.39)
	Posterior	0.30 (0.25)	0.19 (0.29)	0.27 (0.22)	0.33 (0.19)	0.23 (0.29)	0.33 (0.23)	0.34 (0.29)	0.26 (0.25)	0.33 (0.25)	0.44 (0.31)	0.27 (0.31)	0.43 (0.34)

Note. CHI = cognitively healthy individuals, pMCI = possible mild cognitive impairments, naMCI = non-amnestic mild cognitive impairments, aMCI = amnestic mild cognitive impairments. EC = eyes closed, EO = eyes open.

Supplementary Table 2. Log-transformed EC and EO rest absolute power in theta band (4 – 7.5 Hz) as M (SD) for each ROI and group.

		CHI			pMCI			naMCI			aMCI		
		Left	Mid	Right	Left	Mid	Right	Left	Mid	Right	Left	Mid	Right
EC	Frontal	0.33 (0.32)	0.13 (0.39)	0.32 (0.33)	0.30 (0.28)	0.13 (0.33)	0.31 (0.26)	0.22 (0.22)	0.17 (0.27)	0.22 (0.24)	0.34 (0.29)	0.14 (0.34)	0.32 (0.30)
	Central	0.12 (0.35)	-0.03 (0.32)	0.13 (0.35)	0.14 (0.28)	0.03 (0.36)	0.18 (0.37)	0.06 (0.30)	0.01 (0.24)	0.07 (0.27)	0.18 (0.33)	0.06 (0.34)	0.19 (0.36)
	Posterior	0.31 (0.38)	0.08 (0.42)	0.29 (0.40)	0.35 (0.34)	0.12 (0.37)	0.34 (0.37)	0.31 (0.34)	0.07 (0.24)	0.29 (0.33)	0.35 (0.34)	0.08 (0.34)	0.36 (0.36)
EO	Frontal	0.06 (0.27)	-0.06 (0.30)	0.07 (0.26)	0.09 (0.26)	-0.03 (0.30)	0.11 (0.24)	0.12 (0.26)	0.07 (0.24)	0.14 (0.28)	0.18 (0.34)	0.01 (0.32)	0.15 (0.28)
	Central	0.00 (0.28)	-0.18 (0.25)	0.00 (0.29)	0.02 (0.27)	-0.13 (0.31)	0.01 (0.27)	0.04 (0.31)	-0.11 (0.25)	-0.02 (0.26)	0.08 (0.33)	-0.07 (0.31)	0.05 (0.32)
	Posterior	0.12 (0.28)	-0.10 (0.32)	0.07 (0.27)	0.16 (0.25)	-0.07 (0.33)	0.12 (0.28)	0.19 (0.32)	-0.02 (0.31)	0.11 (0.25)	0.19 (0.29)	-0.08 (0.33)	0.16 (0.33)

Note. CHI = cognitively healthy individuals, pMCI = possible mild cognitive impairments, naMCI = non-amnestic mild cognitive impairments, aMCI = amnestic mild cognitive impairments, EC = eyes closed, EO = eyes open.

Supplementary Table 3. Log-transformed EC and EO rest absolute power in alpha band (8 – 13 Hz) as M (SD) for each ROI and group.

		CHI			pMCI			naMCI			aMCI		
		Left	Mid	Right	Left	Mid	Right	Left	Mid	Right	Left	Mid	Right
EC	Frontal	0.63	0.52	0.60	0.66	0.56	0.66	0.54	0.48	0.53	0.69	0.58	0.66
		(0.37)	(0.43)	(0.38)	(0.43)	(0.51)	(0.42)	(0.39)	(0.47)	(0.38)	(0.35)	(0.43)	(0.35)
	Central	0.56	0.32	0.56	0.59	0.36	0.61	0.45	0.29	0.46	0.68	0.46	0.67
		(0.37)	(0.37)	(0.36)	(0.35)	(0.45)	(0.41)	(0.39)	(0.41)	(0.35)	(0.31)	(0.40)	(0.35)
	Posterior	0.90	0.63	0.94	0.94	0.68	0.96	0.80	0.55	0.82	1.00	0.66	1.07
		(0.44)	(0.50)	(0.46)	(0.51)	(0.57)	(0.54)	(0.52)	(0.48)	(0.56)	(0.44)	(0.45)	(0.46)
EO	Frontal	0.27	0.08	0.26	0.31	0.17	0.31	0.27	0.15	0.27	0.34	0.14	0.31
		(0.35)	(0.40)	(0.34)	(0.33)	(0.39)	(0.33)	(0.31)	(0.35)	(0.33)	(0.34)	(0.37)	(0.32)
	Central	0.38	-0.01	0.34	0.40	0.08	0.38	0.34	0.04	0.29	0.46	0.10	0.43
		(0.36)	(0.33)	(0.37)	(0.34)	(0.39)	(0.37)	(0.36)	(0.35)	(0.36)	(0.36)	(0.34)	(0.36)
	Posterior	0.45	0.18	0.42	0.52	0.25	0.49	0.46	0.26	0.39	0.54	0.23	0.53
		(0.38)	(0.46)	(0.39)	(0.38)	(0.45)	(0.40)	(0.39)	(0.42)	(0.39)	(0.36)	(0.40)	(0.37)

Note. CHI = cognitively healthy individuals, pMCI = possible mild cognitive impairments, naMCI = non-amnestic mild cognitive impairments, aMCI = amnestic mild cognitive impairments, EC = eyes closed, EO = eyes open.

Supplementary Table 4. Log-transformed EC and EO rest absolute power in beta band (13.5 – 24 Hz) as M (SD) for each ROI and group.

		CHI			pMCI			naMCI			aMCI		
		Left	Mid	Right	Left	Mid	Right	Left	Mid	Right	Left	Mid	Right
EC	Frontal	0.39 (0.25)	0.25 (0.29)	0.39 (0.26)	0.37 (0.28)	0.27 (0.32)	0.42 (0.29)	0.31 (0.29)	0.24 (0.31)	0.32 (0.31)	0.43 (0.24)	0.28 (0.28)	0.45 (0.23)
	Central	0.45 (0.30)	0.20 (0.30)	0.43 (0.29)	0.45 (0.31)	0.26 (0.38)	0.44 (0.32)	0.29 (0.30)	0.23 (0.38)	0.34 (0.32)	0.48 (0.29)	0.30 (0.31)	0.47 (0.27)
	Posterior	0.51 (0.29)	0.27 (0.31)	0.51 (0.29)	0.52 (0.30)	0.30 (0.33)	0.49 (0.31)	0.40 (0.30)	0.23 (0.30)	0.40 (0.30)	0.54 (0.27)	0.30 (0.29)	0.52 (0.27)
EO	Frontal	0.39 (0.26)	0.19 (0.29)	0.40 (0.29)	0.40 (0.34)	0.22 (0.33)	0.42 (0.30)	0.37 (0.24)	0.23 (0.31)	0.39 (0.30)	0.43 (0.31)	0.22 (0.28)	0.45 (0.29)
	Central	0.49 (0.30)	0.12 (0.30)	0.45 (0.28)	0.43 (0.29)	0.16 (0.39)	0.43 (0.27)	0.39 (0.32)	0.18 (0.40)	0.41 (0.36)	0.45 (0.27)	0.21 (0.33)	0.46 (0.25)
	Posterior	0.48 (0.29)	0.19 (0.31)	0.45 (0.26)	0.47 (0.28)	0.22 (0.35)	0.43 (0.29)	0.41 (0.25)	0.22 (0.29)	0.35 (0.28)	0.48 (0.25)	0.22 (0.32)	0.45 (0.23)

Note. CHI = cognitively healthy individuals, pMCI = possible mild cognitive impairments, naMCI = non-amnestic mild cognitive impairments, aMCI = amnestic mild cognitive impairments, EC = eyes closed, EO = eyes open.

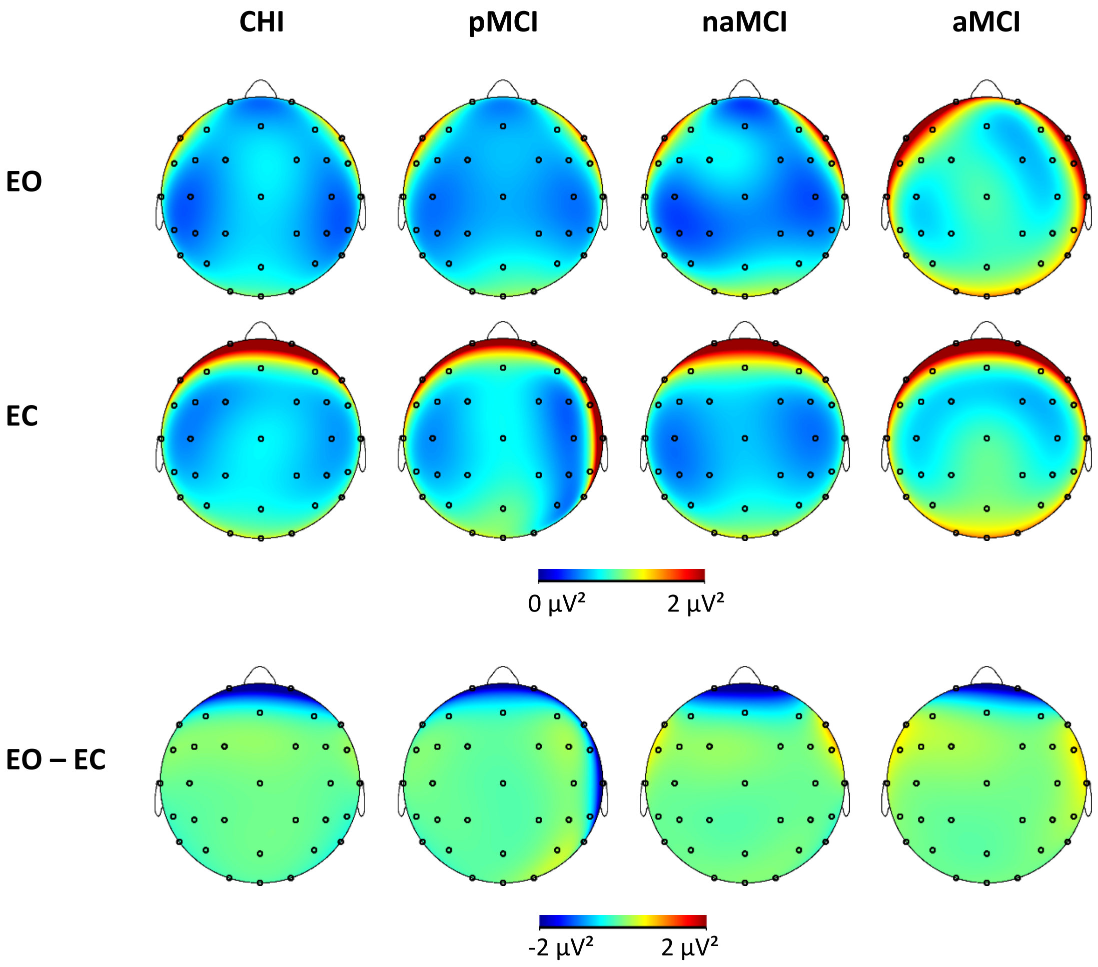

Supplementary Figure 1. Brain maps showing the mean absolute power in µV² (non-transformed) for delta activity (1 – 3.5 Hz) in both conditions and the difference maps separately for each group. CHI = cognitively healthy individuals, pMCI = possible mild cognitive impairments, naMCI = non-amnestic mild cognitive impairments, aMCI = amnestic mild cognitive impairments, EC = eyes closed, EO = eyes open.

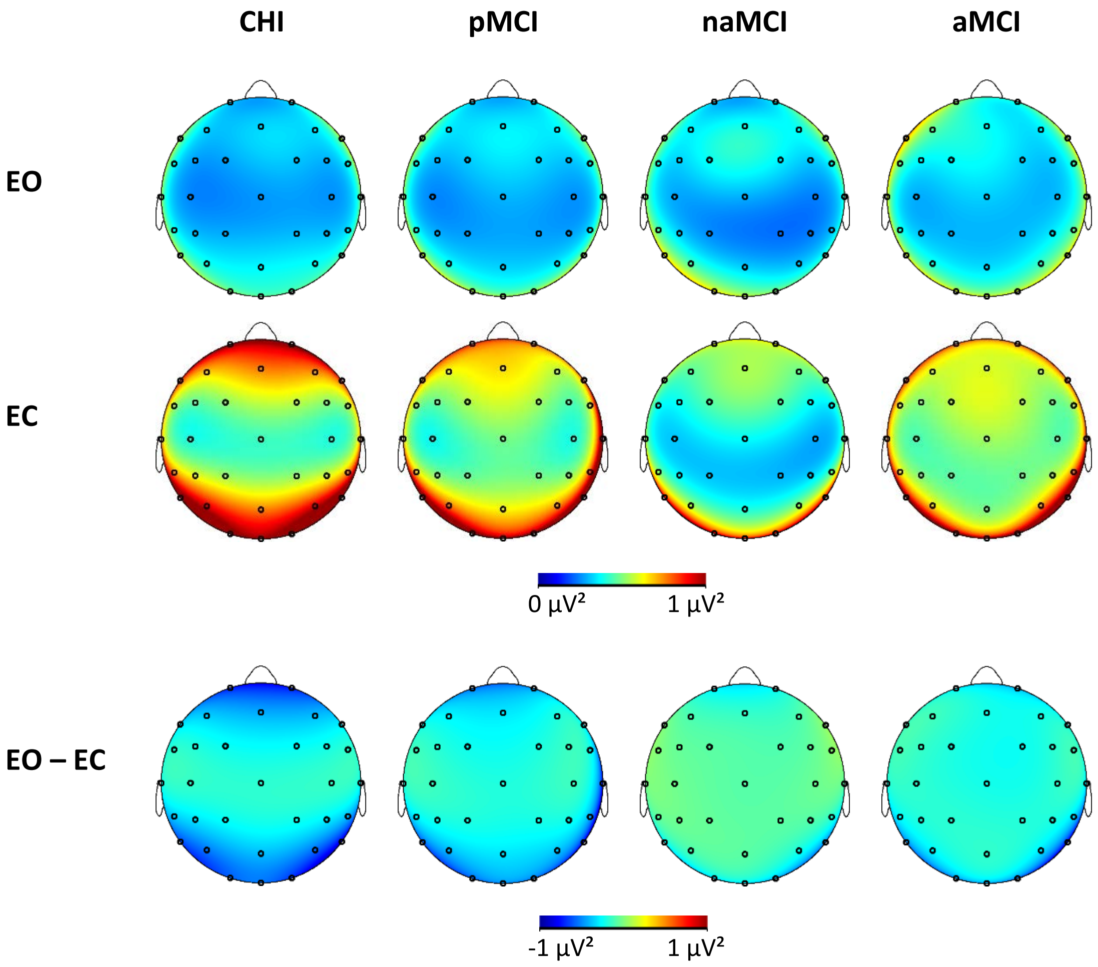

Supplementary Figure 2. Brain maps showing the mean absolute power in µV² (non-transformed) for theta activity (4 – 7.5 Hz) in both conditions and the difference maps separately for each group. CHI = cognitively healthy individuals, pMCI = possible mild cognitive impairments, naMCI = non-amnestic mild cognitive impairments, aMCI = amnestic mild cognitive impairments, EC = eyes closed, EO = eyes open.

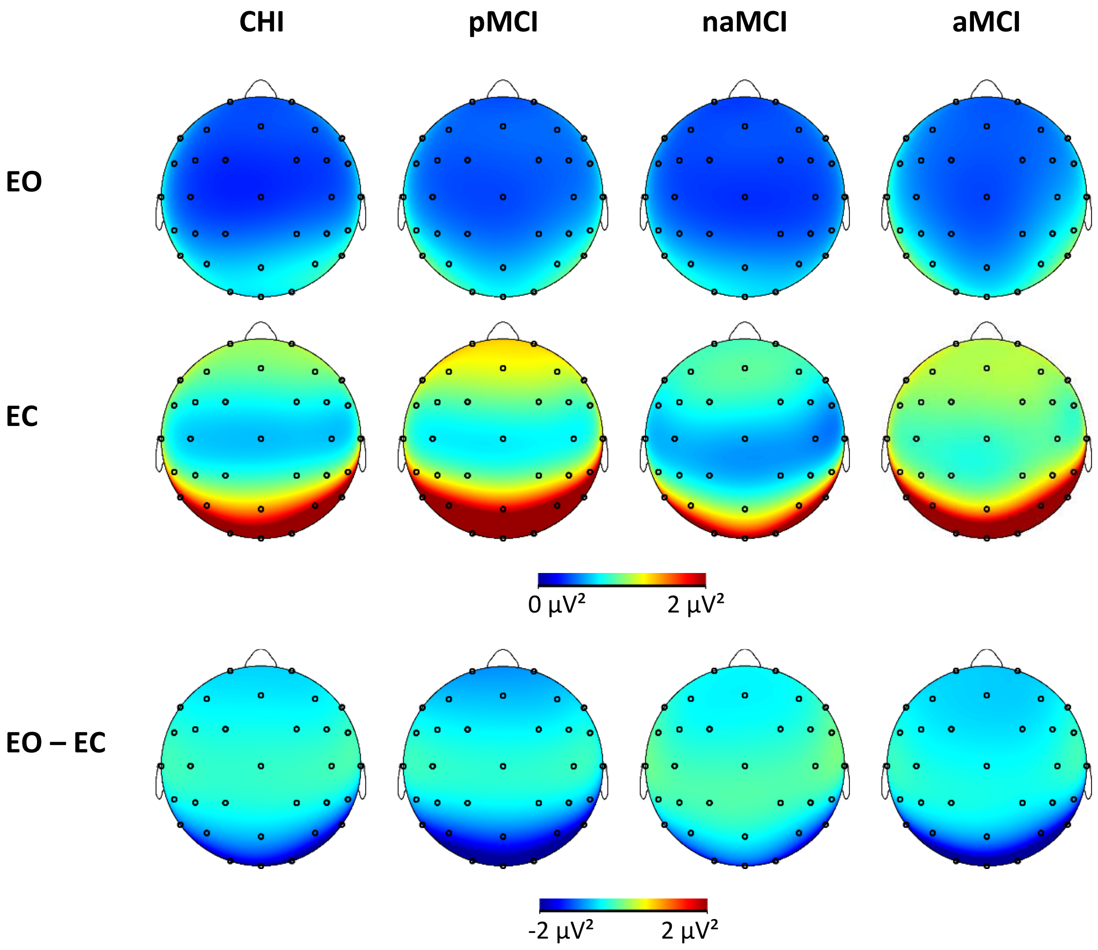

Supplementary Figure 3. Brain maps showing the mean absolute power in µV² (non-transformed) for alpha activity (8 – 13 Hz) in both conditions and the difference maps separately for each group. CHI = cognitively healthy individuals, pMCI = possible mild cognitive impairments, naMCI = non-amnestic mild cognitive impairments, aMCI = amnestic mild cognitive impairments, EC = eyes closed, EO = eyes open.

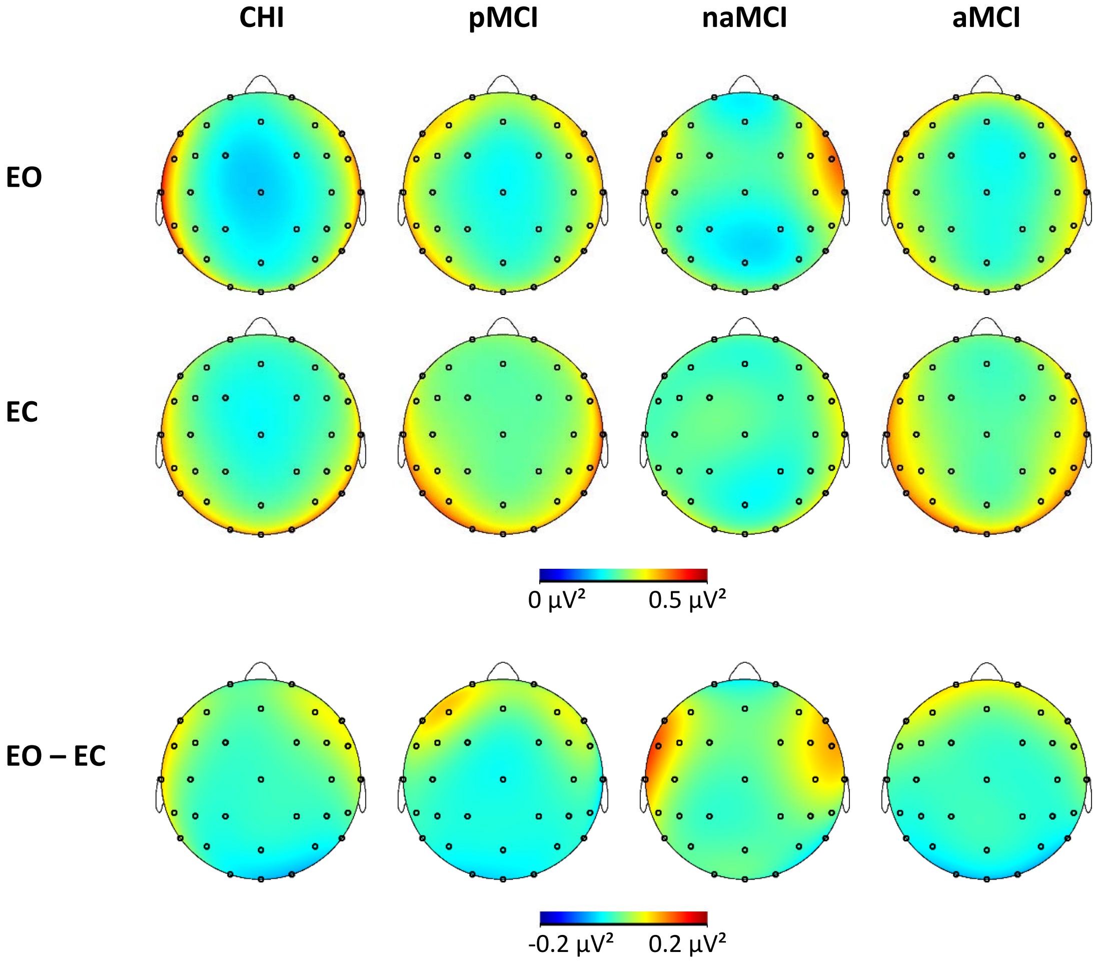

Supplementary Figure 4. Brain maps showing the mean absolute power in µV² (non-transformed) for beta activity (13.5 – 24 Hz) in both conditions and the difference maps separately for each group. CHI = cognitively healthy individuals, pMCI = possible mild cognitive impairments, naMCI = non-amnestic mild cognitive impairments, aMCI = amnestic mild cognitive impairments, EC = eyes closed, EO = eyes open.

Supplementary Table 5. Results of the 3 x 3 x 4 (Coronal x Sagittal x Group) mixed ANCOVA for absolute power (log-transformed) at rest with eyes closed (adjusted α-level = .017).

		DELTA			THETA			ALPHA			BETA		
	df	*F*	*p*	η_p^2	*F*	*p*	η_p^2	*F*	*p*	η_p^2	*F*	*p*	η_p^2
S	(2,414)	206.5	**<.001**	.50	196.4	**<.001**	.49	299.4	**<.001**	.59	106.2	**<.001**	.34
C	(2,414)	133.4	**<.001**	.39	73.3	**<.001**	.26	152.5	**<.001**	.42	13.7	**<.001**	.06
G	(3,207)	0.8	.47	.01	0.3	.84	.00	0.8	.47	.01	0.6	.61	.01
S x C	(4,828)	37.5	**<.001**	.15	4.5	**.003**	.02	34.1	**<.001**	.14	4.6	**.002**	.02
S x G	(6,414)	0.8	.53	.01	1.4	.22	.02	0.7	.62	.01	1.4	.21	.02
C x G	(6,414)	0.6	.71	.01	1.4	.22	.02	1.0	.41	.01	0.4	.49	.01
S x C x G	(12,828)	0.7	.73	.01	1.0	.41	.02	1.6	.14	.02	1.3	.24	.02

Note. Uncorrected degrees of freedom are reported. Sex was included as covariate in the analysis. S = Sagittal, C = Coronal, G = Group.

Supplementary Table 6. Results of the 3 x 3 x 4 (Coronal x Sagittal x Group) mixed ANCOVA for absolute power (log-transformed) at rest with eyes open (adjusted α-level = .017).

		DELTA			THETA			ALPHA			BETA		
	df	*F*	*p*	η_p^2	*F*	*p*	η_p^2	*F*	*p*	η_p^2	*F*	*p*	η_p^2
S	(2,414)	124.7	**<.001**	.38	136.4	**<.001**	.40	318.9	**<.001**	.61	117.0	**<.001**	.36
C	(2,414)	44.6	**<.001**	.18	47.3	**<.001**	.19	50.4	**<.001**	.20	0.8	.43	.00
G	(3,207)	2.7	.05	.04	0.9	.46	.01	0.7	.55	.01	0.2	.90	.00
S x C	(4,828)	9.9	**<.001**	.05	6.7	**<.001**	.03	19.8	**<.001**	.09	3.7	**.01**	.02
S x G	(6,414)	0.8	.56	.01	0.7	.63	.01	1.7	.15	.02	1.3	.27	.02
C x G	(6,414)	0.9	.52	.01	0.0	.48	.01	1.0	.42	.01	0.5	.82	.01
S x C x G	(12,828)	0.9	.50	.01	1.2	.28	.02	0.7	.69	.01	0.8	.59	.01

Note. Uncorrected degrees of freedom are reported. Sex was included as covariate in the analysis. S = Sagittal, C = Coronal, G = Group.

Supplementary Table 7. Results of the 3 x 3 x 4 (Coronal x Sagittal x Group) mixed ANCOVA for absolute power reactivity (log EO – log EC; adjusted α-level = .017).

		DELTA			THETA			ALPHA			BETA		
	df	*F*	*p*	η_p^2	*F*	*p*	η_p^2	*F*	*p*	η_p^2	*F*	*p*	η_p^2
S	(2,412)	2.3	.10	.01	1.3	.28	.01	13.3	**<.001**	.06	11.8	**<.001**	.05
C	(2,412)	23.7	**<.001**	.10	11.4	**<.001**	.05	81.0	**<.001**	.28	12.7	**<.001**	.06
G	(3,206)	1.2	.31	.02	1.9	.13	.03	0.8	.42	.01	1.7	.17	.02
S x C	(4,824)	7.3	**<.001**	.03	3.8	**.01**	.02	21.7	**<.001**	.10	4.0	**.004**	.02
S x G	(6,412)	1.3	.26	.02	0.5	.81	.01	1.4	.23	.02	0.4	.89	.01
C x G	(6,412)	0.4	.89	.01	1.5	.19	.02	0.6	.68	.01	0.8	.56	.01
S x C x G	(12,824)	0.6	.81	.01	0.6	.84	.01	1.3	.26	.02	0.8	.66	.01

Note. Uncorrected degrees of freedom are reported. Sex was included as covariate in the analysis. S = Sagittal, C = Coronal, G = Group.

Supplementary Table 8. Results of the 3 x 3 x 4 (Coronal x Sagittal x Group) mixed ANCOVA for relative power (log-transformed) at rest with eyes closed (adjusted α-level = .017).

		DELTA			THETA			ALPHA			BETA		
	df	*F*	*p*	η_p^2	*F*	*p*	η_p^2	*F*	*p*	η_p^2	*F*	*p*	η_p^2
S	(2,414)	5.9	**.003**	.03	15.1	**<.001**	.07	11.8	**<.001**	.05	2.2	.12	.01
C	(2,414)	138.7	**<.001**	.40	36.2	**<.001**	.15	111.7	**<.001**	.35	81.9	**<.001**	.28
G	(3,207)	0.2	.92	.00	0.4	.76	.01	0.4	.75	.01	0.3	.85	.00
S x C	(4,828)	73.9	**<.001**	.26	3.3	.02	.02	40.9	**<.001**	.17	8.0	**<.001**	.04
S x G	(6,414)	0.7	.62	.01	0.2	.97	.00	1.4	.22	.02	1.4	.24	.02
C x G	(6,414)	1.0	.39	.02	1.1	.36	.02	1.1	.36	.02	0.7	.66	.01
S x C x G	(12,828)	1.0	.44	.01	1.0	.43	.01	0.4	.96	.01	1.7	.07	.02

Note. Uncorrected degrees of freedom are reported. Sex was included as covariate in the analysis. S = Sagittal, C = Coronal, G = Group.

Supplementary Table 9. Results of the 3 x 3 x 4 (Coronal x Sagittal x Group) mixed ANCOVA for relative power (log-transformed) at rest with eyes open (adjusted α-level = .017).

		DELTA			THETA			ALPHA			BETA		
	df	*F*	*p*	η_p^2	*F*	*p*	η_p^2	*F*	*p*	η_p^2	*F*	*p*	η_p^2
S	(2,414)	5.1	**.01**	.02	45.3	**<.001**	.18	17.	**<.001**	.08	3.1	.06	.02
C	(2,414)	40.7	**<.001**	.16	19.9	**<.001**	.09	97.2	**<.001**	.32	12.8	**<.001**	.06
G	(3,207)	0.7	.55	.01	0.6	.59	.01	0.8	.49	.01	0.7	.54	.01
S x C	(4,828)	23.7	**<.001**	.10	22.1	**<.001**	.10	20.1	**<.001**	.09	2.1	.09	.01
S x G	(6,414)	2.1	.07	.03	0.1	.98	.00	0.9	.46	.01	2.6	.03	.04
C x G	(6,414)	0.7	.63	.01	0.3	.94	.00	1.5	.19	.02	0.5	.78	.01
S x C x G	(12,828)	1.6	.09	.02	0.6	.81	.01	0.9	.55	.01	0.9	.49	.01

Note. Uncorrected degrees of freedom are reported. Sex was included as covariate in the analysis. S = Sagittal, C = Coronal, G = Group.

Supplementary Table 10. Results of the 3 x 3 x 4 (Coronal x Sagittal x Group) mixed ANCOVA for relative power reactivity (log EO – log EC; adjusted α-level = .017).

		**DELTA**			**THETA**			**ALPHA**			**BETA**		
	df	*F*	*p*	η_p^2	*F*	*p*	η_p^2	*F*	*p*	η_p^2	*F*	*p*	η_p^2
S	(2,412)	24.3	**<.001**	.11	12.6	**<.001**	.06	3.7	.03	.02	15.2	**<.001**	.07
C	(2,412)	62.8	**<.001**	.23	36.1	**<.001**	.15	21.0	**<.001**	.09	50.3	**<.001**	.20
G	(3,206)	0.5	.68	.01	0.7	.54	.01	0.8	.50	.01	0.7	.54	.01
S x C	(4,824)	20.0	**<.001**	.09	13.2	**<.001**	.06	5.9	**<.001**	.03	3.2	.02	.02
S x G	(6,412)	1.3	.26	.02	0.4	.84	.01	2.8	.02	.04	0.7	.65	.01
C x G	(6,412)	0.5	.79	.01	0.7	.65	.01	1.0	.42	.01	0.9	.47	.01
S x C x G	(12,824)	0.5	.88	.01	0.6	.77	.01	0.4	.92	.01	0.7	.71	.01

Note. Uncorrected degrees of freedom are reported. Sex was included as covariate in the analysis.
S = Sagittal, C = Coronal, G = Group.

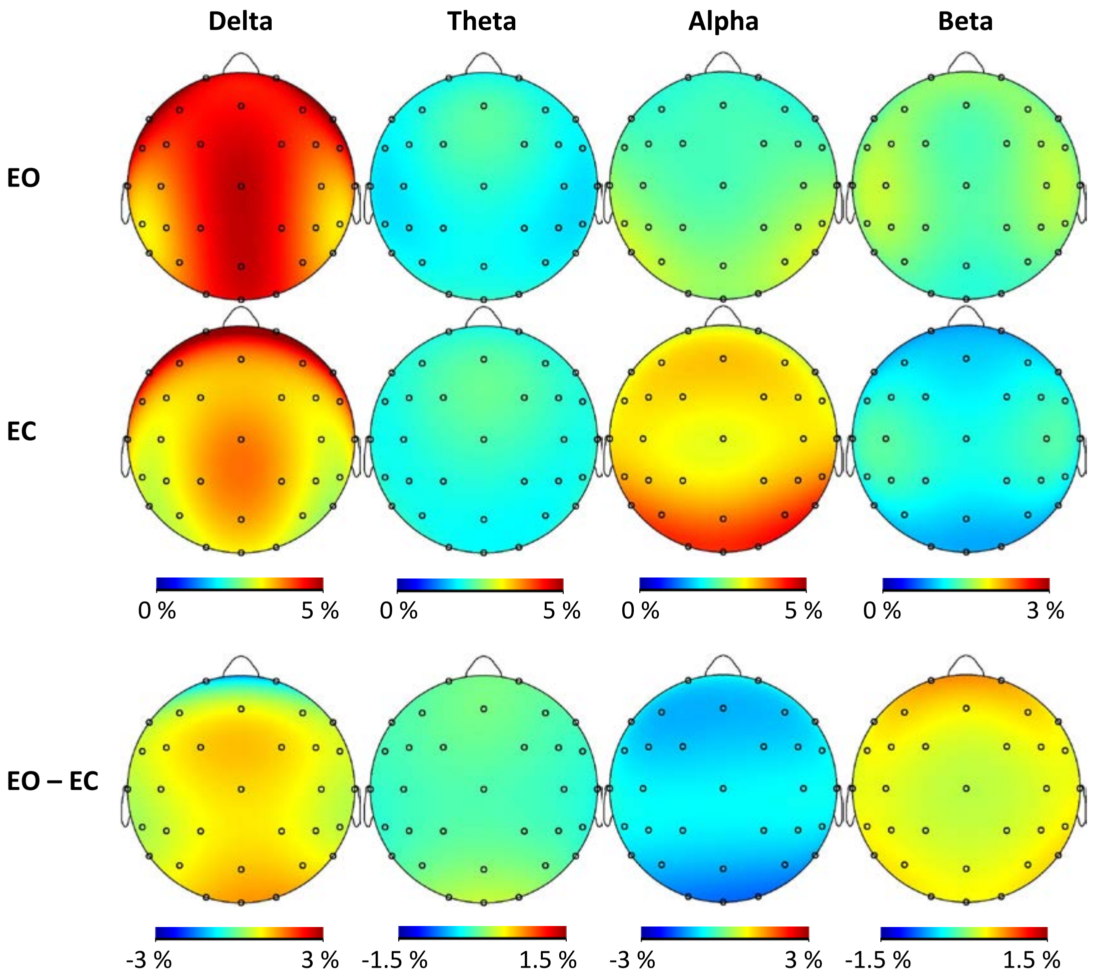

Supplementary Figure 5. Brain maps showing the mean relative power in % for all frequency bands in both conditions and the difference maps. EO = eyes open, EC = eyes closed

Supplementary Table 11. Results of the contrast analysis in each frequency band for relative power (log-transformed) at rest with eyes closed.

	DELTA			THETA			ALPHA			BETA		
	F	*p*	η_p^2	*F*	*p*	η_p^2	*F*	*p*	η_p^2	*F*	*p*	η_p^2
Main Effects (adj. α-level = .025)												
L > R	7.4	**.01**	.04	3.1	.08	.02	4.5	.04	.02			
L/R > M	5.5	**.02**	.03	18.5	**<.001**	.08	13.9	**<.001**	.06			
F > P	325.2	**<.001**	.61	94.4	**<.001**	.31	305.6	**<.001**	.60	0.1	.80	.00
F/P > C	8.7	**.004**	.04	1.4	.24	.01	20.3	**<.001**	.09	161.2	**<.001**	.44
Interactions (adj. α-level = .0125)												
L > R x F > P	0.0	.87	.00				8.5	**.004**	.08	23.0	**<.001**	.10
L > R x F/P > C	1.1	.30	.01				0.0	.99	.00	0.9	.33	.01
L/R > M x F > P	168.9	**<.001**	.45				84.8	**<.001**	.29	0.4	.55	.00
L/R > M x F/P > C	18.6	**<.001**	.08				17.2	**<.001**	.08	14.2	**<.001**	.06

Note. All test statistics are with (1, 207) degrees of freedom. Underlined effects are reversed in direction (i.e., the reversed effect from L > R x F > P is L < R x F > P). Changing the direction of both directional indicators within a single effect is equivalent (i.e., L > R x F > P is the same as L < R x F < P). L = left, R = right, M = midline, F = frontal, P = posterior, C = central.

Supplementary Table 12. Results of the contrast analysis in each frequency band for relative power (log-transformed) at rest with eyes open.

	DELTA			THETA			ALPHA			BETA		
	F	*p*	η_p^2	*F*	*p*	η_p^2	*F*	*p*	η_p^2	*F*	*p*	η_p^2
Main Effects (adj. α-level = .025)												
L > R	0.7	.39	.00	0.4	.54	.00	0.4	.52	.00			
L/R > M	6.4	**.01**	.03	57.4	**<.001**	.22	22.1	**<.001**	.10			
F > P	51.7	**<.001**	.20	10.5	**.001**	.05	190.8	**<.001**	.48	0.9	.35	.00
F/P > C	28.1	**<.001**	.12	28.5	**<.001**	.12	1.6	.20	.01	30.7	**<.001**	.13
Interactions (adj. α-level = .0125)												
L > R x F > P	5.0	.39	.00	0.9	.34	.00	2.6	.11	.01			
L > R x F/P > C	3.1	.08	.02	1.3	.26	.01	1.0	.33	.01			
L/R > M x F > P	46.5	**<.001**	.18	42.8	**<.001**	.17	30.6	**<.001**	.13			
L/R > M x F/P > C	17.7	**<.001**	.08	15.4	**<.001**	.07	25.4	**<.001**	.11			

Note. All test statistics are with (1, 207) degrees of freedom. Underlined effects are reversed in direction (i.e., the reversed effect from L > R x F > P is L < R x F > P). Changing the direction of both directional indicators within a single effect is equivalent (i.e., L > R x F > P is the same as L < R x F < P). L = left, R = right, M = midline, F = frontal, P = posterior, C = central.

Supplementary Table 13. Results of the contrast analysis in each frequency band for reactivity (difference of relative power).

	DELTA			THETA			ALPHA			BETA		
	F	*p*	η_p^2	*F*	*p*	η_p^2	*F*	*p*	η_p^2	*F*	*p*	η_p^2
Main Effects												
L > R	14.0	**<.001**	.06	4.8	.03	.02				0.5	.46	.00
L/R > M	27.5	**<.001**	.12	16.1	**<.001**	.07				20.7	**<.001**	.09
F > P	137.0	**<.001**	.40	39.9	**<.001**	.16	3.9	.05	.02	1.9	.17	.01
F/P > C	0.9	.35	.00	33.7	**<.001**	.14	33.6	**<.001**	.14	84.2	**<.001**	.29
Interactions												
L > R x F > P	4.5	.04	.02	1.0	.32	.01	0.8	.36	.00			
L > R x F/P > C	0.5	.47	.00	5.4	.02	.03	0.8	.38	.00			
L/R > M x F > P	48.9	**<.001**	.19	25.6	**<.001**	.11	13.3	**<.001**	.06			
L/R > M x F/P > C	0.0	.83	.00	5.7	.02	.03	0.7	**.42**	.00			

Note. All test statistics are with (1, 206) degrees of freedom. Underlined effects are reversed in direction (i.e., the reversed effect from L > R x F< P is L < R x F < P). Changing the direction of both directional indicators within a single effect is equivalent (i.e., L > R x F < P is the same as L < R x F > P). L = left, R = right, M = midline, F = frontal, P = posterior, C = central.

Supplementary Table 14. Mean (SD) of absolute reactivity values (log EO – log EC) for the whole sample (N = 211).

	DELTA			THETA			ALPHA			BETA		
	L	M	R	L	M	R	L	M	R	L	M	R
F	-0.24 (0.40)	-0.08 (0.26)	-0.24 (0.39)	-0.21 (0.23)	-0.16 (0.20)	-0.20 (0.23)	-0.35 (0.26)	-0.41 (0.31)	-0.34 (0.26)	**0.02 (0.18)**	-0.05 (0.10)	**0.01 (0.20)**
C	-0.03 (0.21)	-0.04 (0.22)	-0.07 (0.21)	-0.11 (0.14)	-0.15 (0.17)	-0.14 (0.20)	-0.19 (0.17)	-0.31 (0.25)	-0.22 (0.20)	**0.01 (0.20)**	-0.09 (0.12)	**0.01 (0.22)**
P	-0.07 (0.21)	-0.07 (0.21)	-0.08 (0.21)	-0.18 (0.21)	-0.17 (0.21)	-0.21 (0.23)	-0.43 (0.33)	-0.43 (0.34)	-0.50 (0.35)	-0.04 (0.13)	-0.08 (0.13)	-0.06 (0.14)

Note. Positive values are printed in bold and indicate an increase in power from EC to EO. F = frontal, C = central, P =posterior, L = left, M = mid, R = right.
